# Relationship of Depression, Anxiety, and Bipolar Disease with Burning Mouth Syndrome: A Nationwide Cohort Study

**DOI:** 10.3390/ijerph20043391

**Published:** 2023-02-15

**Authors:** Su Jung Lee, Chulho Kim, Hyunjae Yu, Dong-Kyu Kim

**Affiliations:** 1 Research Institute of Nursing Science, School of Nursing, Hallym University, Chuncheon 24252, Republic of Korea; 2 Department of Neurology, Chuncheon Sacred Heart Hospital, Hallym University College of Medicine, Chuncheon 24252, Republic of Korea; 3 Division of Big Data and Artificial Intelligence, Institute of New Frontier Research, Hallym University College of Medicine, Chuncheon 24253, Republic of Korea; 4 Department of Otorhinolaryngology-Head and Neck Surgery, Chuncheon Sacred Heart Hospital, Hallym University College of Medicine, Chuncheon 24253, Republic of Korea

**Keywords:** burning mouth syndrome, depression, anxiety, bipolar, cohort, risk

## Abstract

Burning mouth syndrome (BMS) is a chronic, painful condition of the oral mucosa. Although the pathogenesis remains unclear, psychological and neuroendocrine factors are considered the major contributors. Few longitudinal studies have investigated the effects of psychological factors on the occurrence of BMS. Therefore, we evaluated the risk of BMS in patients with affective disorders using a nationwide population-based cohort dataset. We identified patients with depression, anxiety, and bipolar disorder and then selected comparison participants using the 1:4 propensity score-matching method. We investigated the incidence of BMS events during the follow-up period using survival analysis, the log-rank test, and Cox proportional hazards regression models. After adjusting for other contributing conditions, the adjusted hazard ratio (HR) for developing BMS was 3.37 (95% confidence interval [CI]: 1.67–6.80) for depression and 5.09 (95% CI: 2.19–11.80) for anxiety; however, bipolar disorder showed no significant risk. Specifically, female patients with depression and anxiety had an increased risk of BMS. Moreover, patients with anxiety showed an increased adjusted HR of BMS events during the first 4 years after diagnosis, whereas patients with depression did not. In conclusion, depression and anxiety disorders are significantly associated with the risk of BMS. Additionally, female patients showed a significantly higher risk of BMS than male patients, and anxiety showed increased BMS events earlier than depression. Therefore, clinicians should consider the risk of BMS when treating patients with depression or anxiety.

## 1. Introduction

Burning mouth syndrome (BMS) is a chronic, idiopathic disease characterized by a burning sensation in the oral cavity, with no evident clinical signs and laboratory findings, lasting at least 4–6 months [1]. According to an epidemiologic study, the prevalence of BMS is estimated to be approximately 5% in the general population and it is mainly observed in middle-aged or older adult women [2]. In addition to the burning sensation, many patients also complain of dry mouth (xerostomia) and taste alterations. The diagnostic process for BMS entails thorough and comprehensive history taking and excluding any secondary factors. Physicians should ascertain the patient’s medical and dental history, including past and current symptoms, duration, intensity, character, location, onset, and factors that improve or worsen the pain and its course [3]. Primary BMS is when a burning mouth is not caused by an underlying condition. Meanwhile, secondary BMS is caused by underlying conditions, such as acid reflux; treating the condition often cures burning mouth syndrome. Unfortunately, the specific cause cannot be determined most of the time. Patients with BMS usually experience painful burning sensations in the oral mucosa; they usually self-report annoying, burning, scalding, tingling, and itchy sensations or numbness. In addition to the burning sensation, some patients complain of dry mouth and taste alterations [4,5].

The symptoms of BMS are mostly moderate to severe in intensity and are usually involved in bilateral sides. Although the exact etiology of BMS is currently unknown, it is presumed to be multifactorial, involving interactions between numerous local, systemic, and psychological factors [6]. Previously, several studies reported that psychological comorbidities were common in patients with BMS [7,8,9]. These studies reported that psychologic phenomena, such as alterations in states of anxiety and depression, somatization, and certain aberrant personality traits, are common findings in patients who have BMS. Additionally, a nationwide cohort study reported that BMS is associated with an increased risk of depression and anxiety [10]. Previous studies have also reported that BMS may predispose individuals to depression and anxiety [11,12]. Moreover, one study demonstrated that the severity of BMS symptoms is closely associated with symptoms of psychological distress [13]. However, to our knowledge, no study has examined the risk of BMS in patients with psychological disorders using nationwide population data.

Therefore, we investigated the relationship of BMS with depression, anxiety, and bipolar disorder using a representative sample cohort from the Korea National Insurance Claims dataset. This nationwide population-based dataset allowed us to trace the medical service utilization history of more than one million South Koreans during the long-term period. Moreover, this analysis provided a unique opportunity to examine the incidence and risk of BMS among those diseases, while adjusting for clinical and demographic factors.

## 2. Materials and Methods

We derived the study population from a nationwide representative cohort sample of 1,025,340 adults from the Korea National Insurance Service (KNHIS), spanning a period from 2002 to 2013 accounting for an estimated 2.2% of the South Korean population of approximately 46 million people in 2002. South Korea has had a single-payer national health system covering the entire South Korean population since 1989. An insured individual pays national health insurance proportional to his/her income. Although a user charge exists, it is mandatory for all Koreans to join KNHIS. The KNHIS contains major healthcare information, such as inpatient and outpatient visits, procedures, and prescriptions. Additionally, a unique identification number is assigned to each South Korean at birth, and the KNHIS uses it to identify each South Korean resident. This means that the claims data in the KNHIS could not be omitted or duplicated. Stratified random sampling was performed using 1476 strata; data were categorized by age (18 groups), sex (2 groups), and household income level (41 groups, including 40 health insurance beneficiaries and 1 medical aid beneficiary) among the South Korean population. The health insurance system in South Korea classifies beneficiaries according to income level; the medical aid category comprises lower-income beneficiaries, and the health insurance category comprises higher-income beneficiaries. Therefore, the KNHIS cohort sample can reflect the entire Korean population and minimize the selection bias. The Institutional Review Board (IRB) of Hallym Medical University, Chuncheon Sacred Hospital, approved this retrospective cohort study (IRB No. 2021-08-006). Since this cohort study used de-identified data from a nationwide population-based dataset constructed from national health claims data, the IRB waived the need for written informed consent. The authors confirm that the data supporting the findings of this study are available within the article.

### 2.1. Study Design and Participant Selection

**Figure 1:**
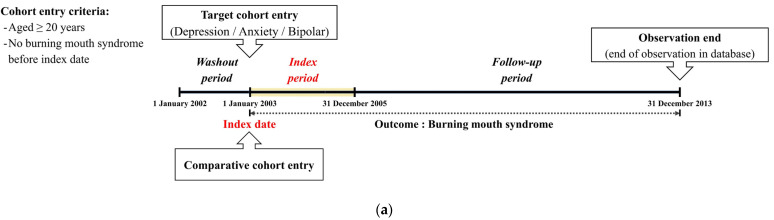

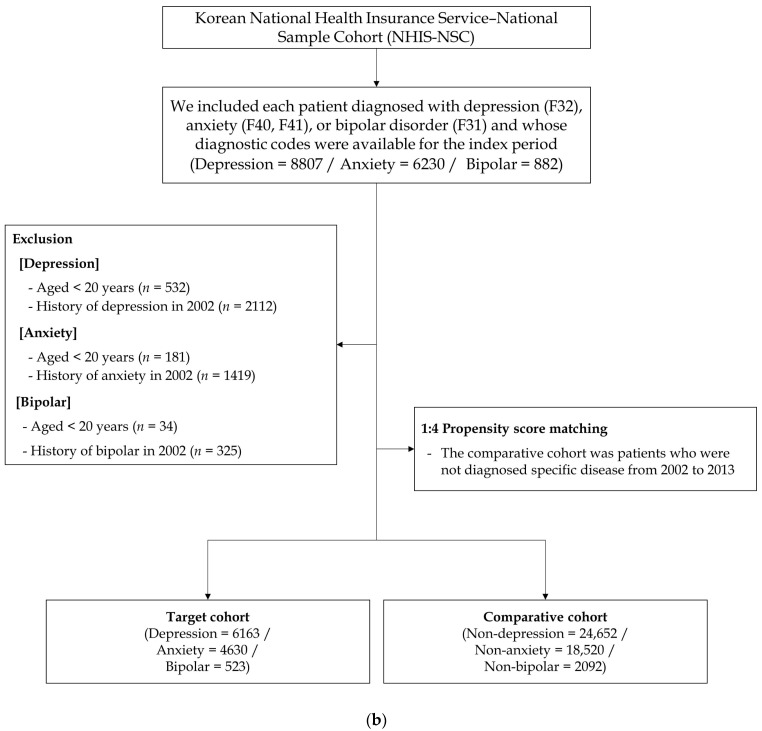
(**a**) Brief description of the study design. (**b**) Flow chart of participant selection.

A brief description of the study design is presented in Figure 1a. This study was designed as a retrospective cohort using healthcare claim data. Thus, we set the washout period, index period, and follow-up period, respectively. Specifically, we conducted a washout period of one year (2002) to remove the possibility of diagnosis for BMS prior to depression, anxiety, and bipolar diagnosis. In this study, we included patients who were diagnosed with depression (F32), anxiety (F40, F41), or bipolar disorder (F31) and whose diagnostic codes were available for the index period. Moreover, we excluded patients (1) aged under 20 years, (2) who died during the index period, or (3) diagnosed with BMS (K14.6) before the diagnosis of depression, anxiety, or bipolar disorder. Additionally, we only selected the diagnostic code for BMS assigned by otorhinolaryngologists to enhance the diagnostic accuracy. Moreover, to eliminate the possibility of including patients who developed BMS before being diagnosed with depression, anxiety, or bipolar disorder, we set the washout period before the index period. Thus, we excluded patients identified in the year 2002 in this database. Then, we selected matched individuals for the comparison group from the remaining cohort registered in the database, namely four participants without cancer for each depression, anxiety, or bipolar patient (Figure 1b).

Age, sex, residence, household income, and comorbidities were considered independent variables. Thus, we controlled for these variables between the two groups in the main analysis, using propensity score matching. Specifically, we obtained information on the comorbidities of each individual and categorized the comorbidities using the Charlson comorbidity index (CCI). It is well-known that comorbidities could influence the development of BMS. Thus, to accurately investigate the effect of affective disorders on the risk of BMS, we controlled for comorbidities using CCI, which is widely used in claims data studies. The CCI is a weighted index used for predicting the risk of death within 1 year of hospitalization for patients with specific comorbid conditions (overall, 19 conditions were included in the index). All independent variables were classified as follows: age (<45, 45–64, >64 years), sex (male and female), residence (Seoul, the largest metropolitan region in South Korea; second area: other metropolitan cities in South Korea; and third area: small cities and rural areas), household income (low: ≤30%, middle: 30.1–69.9%, and high: ≥70% of the median), and comorbidity statuses (CCI: 0, 1, ≥2). A BMS event was the primary endpoint during the follow-up period, and if patients had no events until the final period of this database, we censored this time point (Table 1).

### 2.2. Statistical Analysis 

The overall incidence rate was expressed as per 1000 person-years, and the incidence rate was assessed as a measure of the frequency with which a specific disease or other incident event appeared over a certain period. Cox proportional hazard regression analyses were performed to evaluate whether depression, anxiety, or bipolar disorder could increase the risk of BM by determining the hazard ratio (HR) with 95% confidence intervals (CI). Kaplan–Meier analysis with a log-rank test was performed to identify the differences in specific disease-free (BMS) periods among the study groups. The overall specific disease-free survival rates for the observation period were determined using the Kaplan–Meier survival curve. In this study, the R software program (version 4.0.0; R Foundation for Statistical Computing, Vienna, Austria) was used for all statistical analyses.

## 3. Results

### 3.1. Cohort Sample Characteristics

Our patient cohort comprised one patient each with depression, anxiety, and bipolar disorder per four comparison participants in the comparison cohort (no depression, anxiety, or bipolar disorder). We presented the characteristics of each cohort dataset in Table 2, Table 3 and Table 4.

**Figure 2:**
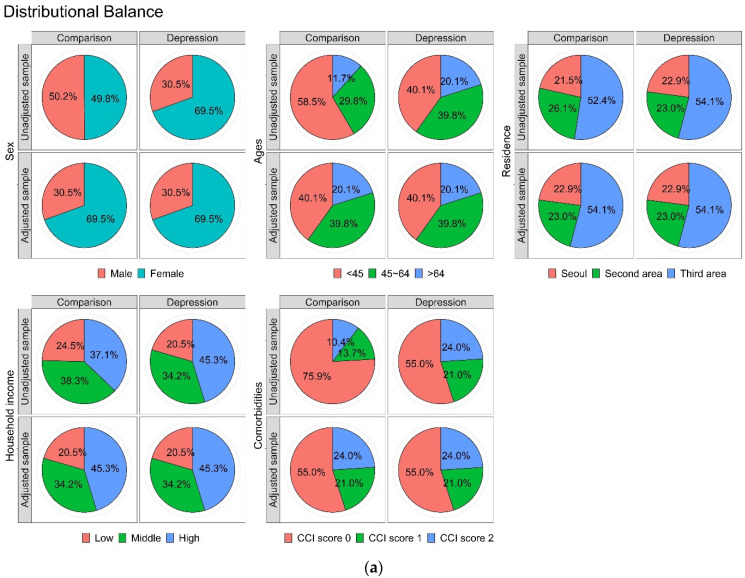

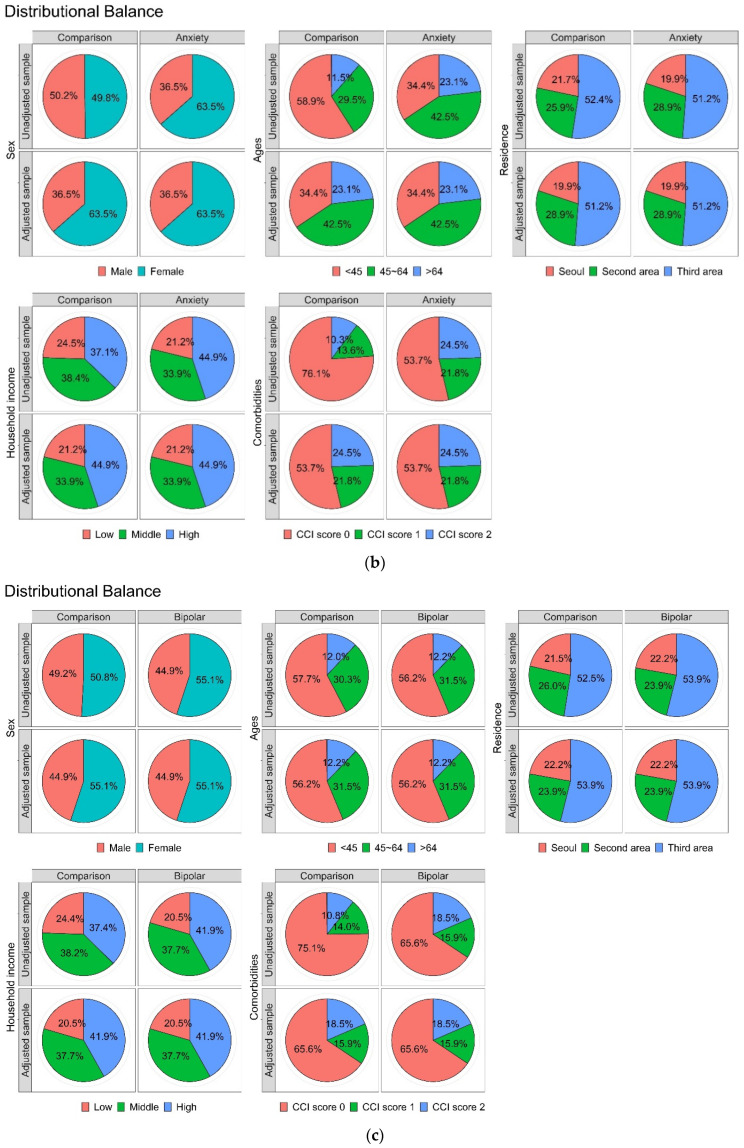
(**a**) Matching of the five main independent variables illustrated by balanced plots between comparison and depression groups. (**b**) Matching of the five main independent variables illustrated by balanced plots between comparison and anxiety groups. (**c**) Matching of the five main independent variables illustrated by balanced plots between comparison and bipolar groups.

There were no significant differences in all independent variables between the two cohorts, indicating that each variable was appropriately matched (Figure 2). In this study, we identified that a total of 228,284.5, 170,302.5, and 19,637.2 person-years in participants with non-depression, non-anxiety, and non-bipolar, respectively, and 54,005.2, 41,005.8, and 4488.6 person-years in patients with depression, anxiety, and bipolar, respectively, were examined in this study. Thus, these cohorts provided a unique opportunity to examine the association between psychological comorbidities (depression, anxiety, and bipolar disorder) and the risk of BMS while adjusting for clinical and demographic factors.

### 3.2. Risk of Subsequent Development of Burning Mouth Syndrome 

The results of univariate and multivariate Cox regression models showed that the adjusted HR for incident BMS events was 3.37 (1.67–6.80) for depression, 5.09 (2.19–11.80) for anxiety, and 3.17 (0.27–37.21) for bipolar disorder (Table 5). 

This result means that patients with depression and anxiety, but not bipolar disorder have a significantly increased risk of developing BMS. In the subgroup analysis, only the female sex showed a significant association with newly developing incident BMS events (adjusted HR = 3.47; 95% CI, 1.56–7.72, and adjusted HR = 4.74; 95% CI, 1.92–11.69, respectively) (Table 6). However, there was no significant difference between patients with BMS and bipolar disorder according to sex. 

**Figure 3:**
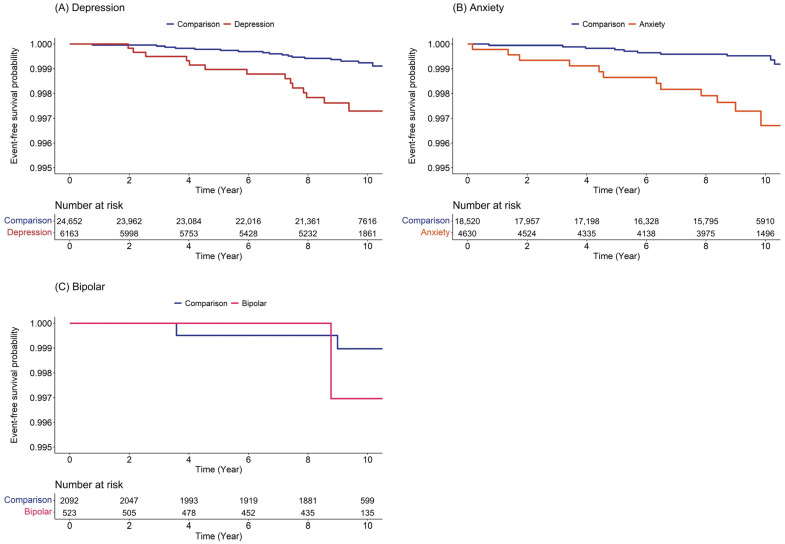
Burning mouth syndrome-free survival probability of burning mouth syndrome in patients with (**A**) depression, (**B**) anxiety, and (**C**) bipolar.

Specifically, the Kaplan–Meier method with a log-rank test showed a more prominent BMS-free survival probability in patients with depression and anxiety than in those with bipolar disorder during the 10-year follow-up period (Figure 3). 

Moreover, we found that the risk of incident BMS events was significantly increased in patients with depression 5 years after the diagnosis, whereas patients with anxiety showed an increased risk of BMS 2 years after the diagnosis of anxiety (Table 7).

## 4. Discussion

The etiology of BMS is presumed to be multifactorial involving the interaction between neurophysiologic and psychologic factors. A considerable number of local, systemic, and psychologic factors have been found related to BMS. It is previously known that BMS is frequently associated with stressful life events, including depression and anxiety, and these psychogenic factors can enhance or reduce the perception of pain. These psychogenic factors also can either enhance or reduce the perception of pain. Several studies have demonstrated that patients with BMS have shown poor scores on all scales that measure quality of life [14,15,16]. However, to date, the precise role of psychological factors in the development of BMS remains controversial [17,18,19]. The pathophysiology of BMS also remains unclear; however, it is most likely the result of multiple factors, including the interaction between psychological and neuropathic factors [3]. Several studies have reported that psychological symptoms, including anxiety, depression, somatization, and certain aberrant personality traits, are commonly found in patients with BMS [17,20]. Another cohort study showed that migraine could increase the risk of developing BMS [21]. However, it is unclear whether psychogenic factors are primary or secondary in any particular case of BMS. Thus, in the long-term retrospective cohort study, we examined the prospective risk of BMS in patients with depression, anxiety, and bipolar disorder, using a nationwide representative sample dataset. 

Interestingly, we observed that patients with depression and anxiety had a higher risk of developing BMS than those without depression and anxiety; however, there was no overall association between bipolar disorder and an increased incidence of BMS. Additionally, our findings revealed that the risk of developing BMS was higher in female patients with depression and anxiety than in male patients. To date, the estimated prevalence of BMS in the general population varies widely in various studies. However, most studies commonly described that females are more likely to have BMS than men, and the prevalence increases with advancing age. Moreover, several epidemiologic studies based on nationwide population showed that BMS affects females more frequently than males [22,23,24]. Some studies have also suggested that altered estrogen levels may contribute to an increased risk of BMS in females [2,25]. These findings are supported by the fact that estrogen receptors have been found not only in the vaginal mucosa but also in the salivary glands in the tongue [26]. One cohort study also demonstrated that postmenopausal patients who received hormone replacement therapy experienced relief from their BMS symptoms [27]. Consistent with these findings, we observed that the risk of incident BMS events was significantly increased only in female patients with depression and anxiety. Additionally, we found that patients with anxiety were at an increased risk of BMS earlier than those with depression. This means that patients with anxiety may be more vulnerable to BMS events than patients with depression. Moreover, we found that the risk of incident BMS development was relatively higher within 5 and 2 years after the diagnosis of depression and anxiety, respectively. At this point, we could not exactly understand why the risk was higher in the early period after those diagnoses; however, it implies to the clinician that careful examination for the early detection of BMS may be useful to prevent the worsening of BMS symptoms in patients with depression or anxiety. Thus, BMS can be managed by pharmacological or psychological means or by combining the two. Consequently, psychological/psychiatric interventions are sometimes considered when BMS does not respond favorably to medication. In particular, cognitive behavioral therapy that helps the patient develop pain-coping strategies was found to be beneficial in reducing suffering.

As with other chronic neuropathic pain conditions, BMS can induce or promote psychic symptoms or can itself be a somatic feature of a psychic disorder. However, it is still unclear whether psychogenic factors are primary or secondary in any particular case of BMS. Previously, we also found that BMS is associated with the increase in subsequent development risk of depression and anxiety, although there was no relationship with dementia or Parkinson’s disease [10]. This study also revealed that female and older patients with BMS showed a significantly higher likelihood of incident depression and anxiety events. In addition to psychogenic factors, both peripheral and central neuropathies also play a role in the development of BMS, but the balance between central and peripheral neuropathies may vary according to the patients with BMS. It is considered that the difference between genetic and environmental factors contributes to determining individual differences in the experience of pain. Our previous study also found that patients with migraine were associated with an increased risk of BMS events compared to those without migraines. This study could support that damage to peripheral small nerve fibers in migraine patients may result in clinical BMS presentations often described as burning, tingling, and numbness [21]. 

To date, in terms of BMS treatment, there is no cure, and it is purely symptomatic care; thus, expectations of the outcome of BMS treatment should not be unrealistic. For these reasons, there is no standard treatment, and it is empirical and largely based on personal and expert opinions. The use of both topical and systemic clonazepam has been reported to reduce the intensity of the pain of BMS. Topical capsaicin is also used because it causes reversible degeneration of peripheral sensory nerve endings, with the consequent reduction in the syndromal burning pain sensation. Antidepressants are often used to treat BMS most probably because of their documented effects in reducing the intensity of neuropathic pain and because of the close association between BMS and generalized anxiety and depressive disorders. Cognitive behavioral therapy may be helpful to BMS patients. With cognitive behavioral therapy, harmful thoughts, and problematic behaviors are identified, and the dysfunctional relationship between those could be explained to the patient. If BMS patients could understand the mechanisms that drive dysregulated relationships between thought and behavior, their levels of depression and anxiety may decrease.

In this long-term retrospective cohort study, we also could not conclude whether this association was a possible link between the two diseases or an incidental temporal finding. However, this nationwide population-based dataset allowed us to identify the total medical service history; hence, we could assess the association between psychological comorbidities and the risk of BMS while adjusting for clinical and demographic factors. Numerous retrospective cohort studies have already published similar study designs using this database [28,29,30,31,32,33]. Moreover, we selected patients with BMS diagnosed by otorhinolaryngologists to improve diagnostic accuracy. Finally, we minimized the surveillance bias on the risk of BMS in depression, anxiety, and bipolar patients because we selected sociodemographic characteristics that matched controls in the cohort database. Moreover, our database could effectively analyze all event relationships because our retrospective cohort study had a long follow-up period and could represent the South Korean population. This study had some notable limitations. First, we could not control for specific behavioral information, such as smoking and alcohol consumption; these factors could contribute to the subsequent development of BMS as local risk factors. Second, psychological comorbidities usually tend to be underdiagnosed, and depression and anxiety also include a wide variety of disease states that cannot be distinguished, depending on the phenotype of the disease, given the somewhat simplistic diagnostic code system used here. Thus, disease definitions based on the diagnostic code are limited in their evaluations of the association between given diseases. Third, we could not control medication for psychological comorbidities. These factors may influence the severity of BMS symptoms and may be related to the initial BMS diagnosis. Fourth, it is known that to identify the etiology of BMS, imaging data of the brain via CT or MRI in BMS patients is helpful to determine whether sensory and/or motor disturbances, autonomic changes, or any other evidence suggestive of the neurodegenerative process exist. The salivary structures image in BMS patients is also helpful to be identified BMS etiology. However, in this study, we could not access all imaging data. Finally, this was a retrospective cohort study; thus, we could not directly examine the pathological mechanisms underlying the relationship between the conditions. Therefore, further experimental studies are needed in this regard.

## 5. Conclusions

In the long-term retrospective cohort study, we examined whether patients with depression, anxiety, and bipolar disorder had an increased risk of developing BMS using a nationwide population database based on the nationwide cohort dataset. Our findings suggest that depression and anxiety, but not bipolar disorder are closely related to the development of BMS. Additionally, female patients showed a significantly higher risk of developing BMS than male patients, and patients with anxiety had an increased risk of incident BMS events earlier than patients with depression. Therefore, in light of our results, clinicians should be aware of the risk of BMS in patients with depression and anxiety. 

## Figures and Tables

**Table 1:** Description of time to event and censored data.

	BMS Event (Depression)	BMS Event (Anxiety)	BMS Event (Bipolar)
Event	33	22	3
Comparison	19	10	2
Depression, anxiety, or bipolar	14	12	1
Total censored (no event)	30,782	23,128	2612
Comparison	24,633	18,510	2090
Depression, anxiety, or bipolar	6149	4618	522
Termination of study	26,025	19,298	2281
Comparison	20,916	15,402	1858
Depression, anxiety, or bipolar	5109	3896	423
Loss to follow-up/drop-out	4757	3830	331
Comparison	3717	3108	232
Depression, anxiety, or bipolar	1040	722	99

BMS; burning mouth syndrome.

**Table 2:** Characteristics of the enrolled participants in depression: comparison vs. depression.

Variables	Comparison (n = 24,652)	Depression (n = 6163)	*p* Value
Sex			1.000
Male	7524 (30.5%)	1881 (30.5%)	
Female	17,128 (69.5%)	4282 (69.5%)	
Ages (years)			1.000
<45	9896 (40.1%)	2474 (40.1%)	
45–64	9804 (39.8%)	2451 (39.8%)	
>64	4952 (20.1%)	1238 (20.1%)	
Residence			1.000
Seoul	5648 (22.9%)	1412 (22.9%)	
Second area	5676 (23.0%)	1419 (23.0%)	
Third area	13,328 (54.1%)	3332 (54.1%)	
Household income			1.000
Low (0–30%)	5060 (20.5%)	1265 (20.5%)	
Middle (30–70%)	8420 (34.2%)	2105 (34.2%)	
High (70–100%)	11,172 (45.3%)	2793 (45.3%)	
CCI			1.000
0	13,568 (55.0%)	3392 (55.0%)	
1	5168 (21.0%)	1292 (21.0%)	
≥2	5916 (24.0%)	1479 (24.0%)	

Comparison, subjects without depression; Seoul, the largest metropolitan area; second area, other metropolitan cities; third area, other areas; CCI, Charlson comorbidity index.

**Table 3:** Characteristics of the enrolled participants: comparison vs. anxiety group.

Variables	Comparison (n = 18,520)	Anxiety (n = 4630)	*p* Value
Sex			1.000
Male	6756 (36.5%)	1689 (36.5%)	
Female	11,764 (63.5%)	2941 (63.5%)	
Ages (years)			1.000
<45	6372 (34.4%)	1593 (34.4%)	
45–64	7872 (42.5%)	1968 (42.5%)	
>64	4276 (23.1%)	1069 (23.1%)	
Residence			1.000
Seoul	3680 (19.9%)	920 (19.9%)	
Second area	5360 (28.9%)	1340 (28.9%)	
Third area	9480 (51.2%)	2370 (51.2%)	
Household income			1.000
Low (0–30%)	3924 (21.2%)	981 (21.2%)	
Middle (30–70%)	6284 (33.9%)	1571 (33.9%)	
High (70–100%)	8312 (44.9%)	2078 (44.9%)	
CCI			1.000
0	9952 (53.7%)	2488 (53.7%)	
1	4032 (21.8%)	1008 (21.8%)	
≥2	4536 (24.5%)	1134 (24.5%)	

Comparison, subjects without anxiety; Seoul, the largest metropolitan area; second area, other metropolitan cities; third area, other areas; CCI, Charlson comorbidity index.

**Table 4:** Characteristics of the enrolled participants: comparison vs. bipolar group.

Variables	Comparison (n = 2092)	Bipolar (n = 523)	*p* Value
Sex			1.000
Male	940 (44.9%)	235 (44.9%)	
Female	1152 (55.1%)	288 (55.1%)	
Ages (years)			1.000
<45	1176 (56.2%)	294 (56.2%)	
45–64	660 (31.5%)	165 (31.5%)	
>64	256 (12.2%)	64 (12.2%)	
Residence			1.000
Seoul	464 (22.2%)	116 (22.2%)	
Second area	500 (23.9%)	125 (23.9%)	
Third area	1128 (53.9%)	282 (53.9%)	
Household income			1.000
Low (0–30%)	428 (20.5%)	107 (20.5%)	
Middle (30–70%)	788 (37.7%)	197 (37.7%)	
High (70–100%)	876 (41.9%)	219 (41.9%)	
CCI			1.000
0	1372 (65.6%)	343 (65.6%)	
1	332 (15.9%)	83 (15.9%)	
≥2	388 (18.5%)	97 (18.5%)	

Comparison, subjects without bipolar disorder; Seoul, the largest metropolitan area; second area, other metropolitan cities; third area, other areas; CCI, Charlson comorbidity index.

**Table 5:** The risk of incident burning mouth syndrome in patients with depression, anxiety, and bipolar compared to that in the comparison group.

Variables	N	Case	Person-Years	Incidence Rate	Unadjusted HR (95% CI)	Adjusted HR (95% CI)
Comparison	24,652	19	228,284.5	0.08	1.00 (ref)	1.00 (ref)
Depression	6163	14	54,005.2	0.26	3.40 (1.69–6.87) ***	3.37 (1.67–6.80) ***
Comparison	18,520	10	170,302.5	0.06	1.00 (ref)	1.00 (ref)
Anxiety	4630	12	41,005.8	0.29	5.14 (2.22–11.91) ***	5.09 (2.19–11.80) ***
Comparison	2092	2	19,637.2	0.10	1.00 (ref)	1.00 (ref)
Bipolar	523	1	4488.6	0.22	2.66 (0.24–29.67)	3.17 (0.27–37.21)

HR, hazard ratio; CI, confidence interval. *** *p* < 0.001.

**Table 6:** Hazard ratios of incident burning mouth syndrome by sex in patients with depression or anxiety.

Sex	Male		Female	
	Comparison	Depression	Comparison	Depression
**Depression**				
Unadjusted HR (95% CI)	1.00 (ref)	3.05 (0.70–13.33)	1.00 (ref)	3.50 (1.57–7.80) **
Adjusted HR (95% CI)	1.00 (ref)	3.08 (0.71–13.46)	1.00 (ref)	3.47 (1.5–67.72) **
**Anxiety**				
Unadjusted HR (95% CI)	1.00 (ref)	7.92 (0.72–87.35)	1.00 (ref)	4.80 (1.95–11.85) ***
Adjusted HR (95% CI)	1.00 (ref)	7.71 (0.70–85.11)	1.00 (ref)	4.74 (1.92–11.69) ***

HR, hazard ratio; CI, confidence interval. NA: non-applicable ** *p* < 0.010, and *** *p* < 0.001.

**Table 7:** Risk ratio of burning mouth syndrome event by time elapsed since the diagnosis of depression or anxiety.

Time (Year) after Diagnosis	Depression	Anxiety
	Adjusted HR (95% CI)	Adjusted HR (95% CI)
1	0.00 (0–Inf)	3.96 (0.25–63.28)
2	4.02 (0.25–64.26)	11.85 (1.23–113.91) *
3	5.97 (1.00–35.75)	11.85 (1.23–113.91) *
4	3.96 (0.99–15.82)	5.34 (1.19–23.86) *
5	4.78 (1.46–15.67) **	6.00 (1.69–21.27) **
6	3.98 (1.40–11.34) **	3.97 (1.28–12.32) *
7	3.10 (1.15–8.32) *	4.54 (1.65–12.53) **
8	3.72 (1.70–8.15) **	5.11 (1.90–13.72) **
9	3.80 (1.79–8.09) ***	5.62 (2.26–13.97) ***
10	3.64 (1.78–7.47) ***	6.26 (2.55–15.33) ***
11	3.37 (1.67–6.80) ***	5.09 (2.19–11.80) ***

HR, hazard ratio; CI, confidence interval. * *p* < 0.05, ** *p* < 0.010, and *** *p* < 0.001.

## Data Availability

The authors confirm that the data supporting the findings of this study are available within the article.
